# Supplementary material for: Time‑resolved multi-omic analysis of paclitaxel exposure in human iPSC‑derived sensory neurons unveils mechanisms of chemotherapy‑induced peripheral neuropathy
Source: Cell Death Dis. 2026 Feb 10;17(1):211. doi: 10.1038/s41419-026-08445-2 (PMC12921266; doi:10.1038/s41419-026-08445-2)
Supplement: Supplementary file 15 — Dataset 1. [file 41419_2026_8445_MOESM15_ESM.zip › CDD_Open_Data/04_RNA_Seq/Timeline_panel_plots_etc_2023-01-23_1609.zip!/Panel_Plots_etc/Evidence_Plots/post5d/evidence_plot_standard contrasts_msigdb_reactome_5d_PTX_vs_DMSO_ID6_M27455_REACTOME_PHASE_0_RAPID_DEPOLARISATION.pdf]

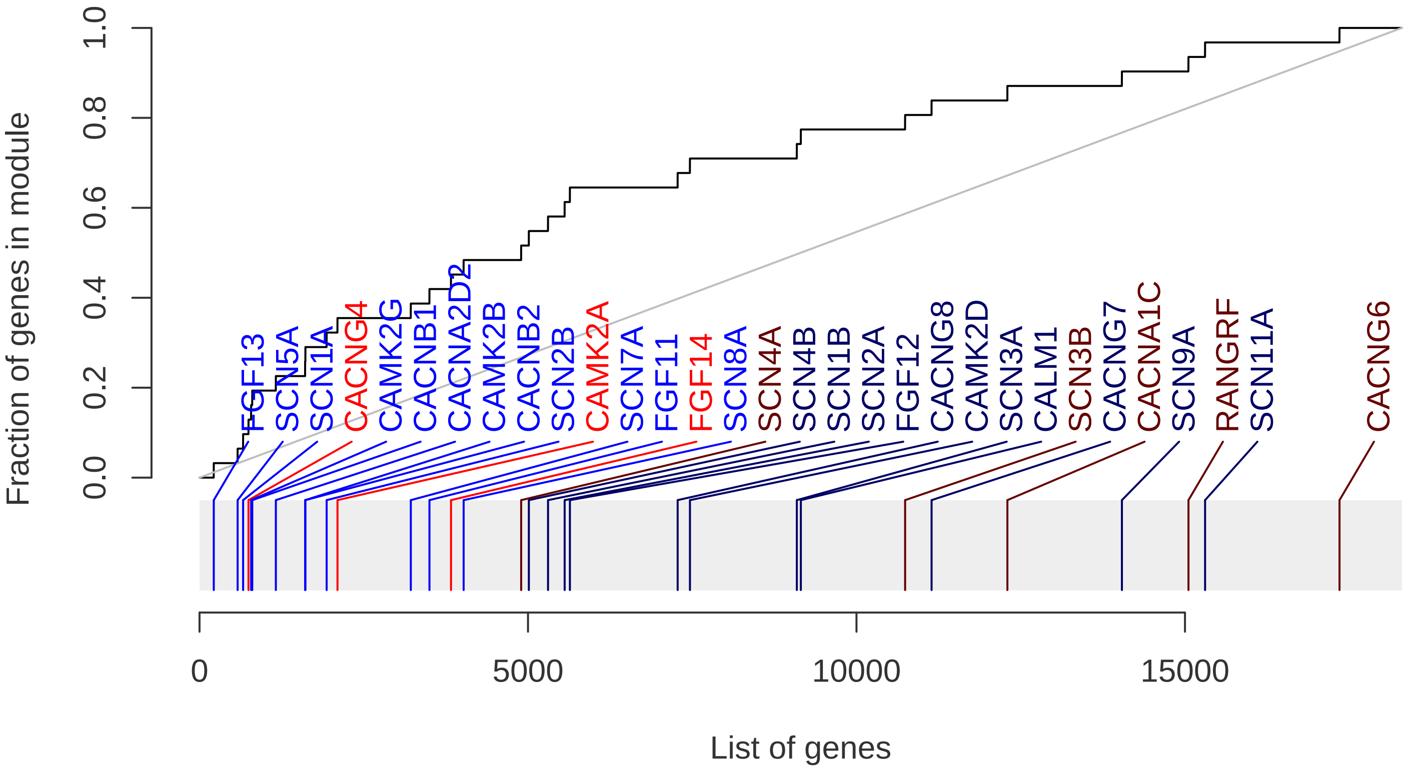

Fraction of genes in module
0.0
0.2
0.4
0.6
0.8
1.0
FGF13
SCN5A
SCN1A
CACNG4
CAMK2G
CACNB1
CACNA2D2
CAMK2B
CACNB2
SCN2B
CAMK2A
SCN7A
FGF11
FGF14
SCN8A
SCN4A
SCN4B
SCN1B
SCN2A
FGF12
CACNG8
CAMK2D
SCN3A
CALM1
SCN3B
CACNG7
CACNA1C
SCN9A
RANGRF
SCN11A
CACNG6
0
5000
10000
15000
List of genes
